# Supplementary material for: Phased chromosome-scale genome assembly of an asexual, allopolyploid root-knot nematode reveals complex subgenomic structure
Source: PLoS One. 2024 Jun 6;19(6):e0302506. doi: 10.1371/journal.pone.0302506 (PMC11156385; doi:10.1371/journal.pone.0302506)
Supplement: S1 File — (PDF) [file pone.0302506.s001.pdf]

Supplementary methods

DNA extraction and sequencing

High molecular weight DNA isolation

Two ml of lysis buffer containing 100mM NaCl, 10 mM Tris-HCl pH 8.0, 25 mM EDTA, 0.5% (w/v) SDS and 100μg/ml Proteinase K was added to the tube containing ~1,000,000 flash frozen eggs. Samples were mixed with gentle pipetting and homogenised at room temperature overnight. Lysate was then treated with 20μg/ml RNAse at 370C for 30 minutes. The lysate was cleaned with equal volumes of phenol/chloroform using phase lock gels (Quantabio Cat # 2302830). The DNA was precipitated from the cleaned lysate by adding 0.4X volume of 5M ammonium acetate and 3X volume of ice-cold ethanol. The DNA pellet was washed with 70% ethanol twice and resuspended in an elution buffer (10mM Tris, pH 8.0). Purity of gDNA was accessed using NanoDrop ND-1000 spectrophotometer and 260/280 ratio of 1.9 and 260/230 of 2.29 were observed. DNA yield was quantified using Qubit 2.0 Fluorometer (Thermofisher Scientific, MA).

PacBio HiFi

All PacBio HiFi libraries were prepared the following way. HiFi SMRTbell libraries were constructed using the SMRTbell Express Template Prep Kit v2.0 (Pacific Biosciences, Menlo Park, CA; Cat. #100-938-900) according to the manufacturer's instructions. HMW gDNA was sheared to a target DNA size distribution between 15 kb – 20 kb using Diagenode's Megaruptor 3 system (Diagenode, Belgium; Cat. B06010003). The sheared gDNA was concentrated using 0.45X of AMPure PB beads (Pacific Biosciences, Menlo Park, CA; Cat. #100-265-900) for the removal of single-strand overhangs at 37 °C for 15 minutes, followed by further enzymatic steps of DNA damage repair at 37 °C for 30

minutes, end repair and A-tailing at 20 °C for 10 minutes and 65 °C for 30 minutes, ligation of barcoded overhang adapters v3 at 20 °C for 60 minutes and 65 °C for 10 minutes to inactivate the ligase, then nuclease treated at 37 °C for 1 hour. SMRTbell libraries were purified and concentrated with 0.45X Ampure PB beads for size selection using the BluePippin/PippinHT system (Sage Science, Beverly, MA; Cat #BLF7510/HPE7510) to collect fragments greater than 7-9 kb. HiFi SMRTbell libraries were sequenced at UC Davis DNA Technologies Core (Davis, CA) using one SMRT® Cell 8M Tray (Pacific Biosciences, Menlo Park, CA; Cat #101-389-001), Sequel II sequencing chemistry 2.0, and 30-hour movies each on a PacBio Sequel II sequencer.

PacBio Iso-Seq

cDNA synthesis was prepared using NEBNext Single Cell/Low Input cDNA Synthesis & Amplification Module kit (New England Biolabs Inc., Ipswich, MA; Cat #E6421L) according to manufacturer's instructions with a slight modification to the PCR cycles of 15 cycles. Amplified cDNA samples were purified using 0.86X of ProNex beads (Promega, Madison, WI; Cat #NG2003) for equal molar pooling of barcoded cDNA. The pooled cDNA was constructed into the SMRTbell Express Template Prep Kit v2.0 (Pacific Biosciences, Menlo Park, CA; Cat. #100-938-900) according to the manufacturer's instructions. The enzymatic steps included DNA damage repair at 37 °C for 30 minutes, end repair and A-tailing at 20 °C for 30 minutes and 65 °C for 30 minutes, and ligation of adapters v3 at 20 °C for 60 minutes. The SMRTbell library was purified with 1X ProNex beads. The Iso-Seq SMRTbell library was sequenced at UC Davis DNA Technologies Core (Davis, CA) using one SMRT® Cell 8M Tray (Pacific Biosciences, Menlo Park, CA; Cat #101-389-001), Sequel II sequencing chemistry 2.0, and 30-hour movies each on a PacBio Sequel II sequencer.

Oxford Nanopore

A sequencing library was prepared starting with 2μg of gDNA using the ligation sequencing kit SQK-LSK109 (Oxford Nanopore Technologies, Oxford, UK) following instructions of the manufacturer except for extended incubation times for DNA damage repair, end repair, ligation, and bead elutions. 30 fmol of the final library was loaded on the PromethION flow cell R9.4.1 (Oxford Nanopore Technologies, Oxford, UK) and the data was collected for seventy-two hours. Base calling was performed real-time on the PromethION compute tower using MinKNOW 20.06.9 and guppy v4.0

Draft assemblies

Draft assemblies were generated with different assemblers to discern which assembly method or package would perform better with our data. We applied *Canu*, *FALCON*, *Shasta*, *IPA*, and *HiFiasm* with iteratively differing parameters [74-78]. All assemblers barring *HiFiasm* produced highly fragmented assemblies in comparison, and *HiFiasm* was chosen as the best applicable assembly method. Using *HiFiasm* and iterating through different parameters, we produced upwards of fifty draft assemblies, of which the primary assembly generated during our fifteenth parameter iteration was deemed the best for downstream analysis.

Assembly appraisal

All the following analyses and assessments were performed as part of the *asmapp* workflow [79], a genome assembly appraisal workflow that automates these processes.

Mitochondrial detection and analysis

The mitochondrial genome was detected using *BLAST* [92], querying a previously published *M. javanica* mitochondrial genome (ACC: NC_026556.1) against a database generated from our assembly. Mitochondrial annotation was performed using the *MITOS* web server [93].

Core Gene Analysis

A core gene presence and completeness analysis was performed by both *CEGMA* [42] and *BUSCOv5* [88]. *CEGMA* was used with its default settings. *BUSCOv5* was used with the eukaryota_odb10 database. We chose to use the eukaryote dataset due to the lack of *Meloidogyne* representation in the nematode *BUSCO* dataset, although analysis with nematoda_odb10 was also performed (Supplementary Table 1).

Contamination analysis

Contaminants were detected using *Blobtools* [41]. *Blobtools* uses GC%, coverage, and taxonomic assignment to identify contaminant contigs within the assembly. Taxonomy was assigned based on Phylum level matches to the complete NCBI BLAST nucleotide database.

Coverage

Coverage information was generated using *samtools coverage* [94]. Plots of coverage are generated from the samtools coverage output using several custom R scripts.

QUAST

Full descriptive statistics of all assemblies were generated using *QUAST* [95].

Annotation

Repeat annotation

A repeat model library was generated by *RepeatModeler* [83]. This library was then used to inform *RepeatMasker* to identify and mask repeats in the genome [84].

Iso-Seq

Following sequencing, Iso-Seq reads were provided as full-length non-chimeric sequences which were transformed, mapped to the assembly, and collapsed using the *IsoSeq3* pipeline from Pacific Biosciences with default settings [73].

MAKER3

MAKER3 was used to perform prediction-based annotations [85]. An initial run was performed using *MAKER3*'s *Exonorate* module, informed by previously published *M. javanica* annotations [19] and the Iso-Seq transcriptional library we generated here. Following this, annotations were validated by fathom, and a second *MAKER3* run was initiated using the *SNAP* module. Annotations were then validated based on annotation edit distance (AED) and extracted as .gff and .fasta.

Pairs analysis

Orthology

Candidate homoeologous pairs were identified through shared CDS. Iso-Seq transcripts were queried against the assembly using *BLAST* to find orthologs throughout the assembly, with coordinates and counts of matching pairs recorded. Hits were only counted over 97% similarity.

Duplicated core genes

Scaffolds that shared more duplicated BUSCO genes than expected by chance were designated as potential homoeologous pairs. BUSCO genes are represented in a single copy, per copy. Scaffold pairs that share duplicates of the same BUSCO genes are likely homoeologous.

MASH distance

Candidate pairs were also identified using *MASH* hash mapping [87]. A sketch was created of the genome and scaffolds were iteratively compared against it. Non-zero MASH similarity scores to non-self scaffolds suggest sequence similarity and homology.

Nucleotide similarity

Scaffolds that share regions of high similarity are potentially homoeologous. We used a combination of *BLAST* and *Nucmer* to identify for each scaffold in the assembly which other non-self scaffold exhibited the highest level of nucleotide similarity [96].

Phasing subgenomes

Scaffolds identified as present in homoeologous pairs were phased using a method based on that used by Cerca et al (2021) [45]. This method works on the logic that in a hybrid we would expect to find k-mers unique to each subgenomes' lineage, resulting from genomic divergence accumulated since speciation from a common ancestor. Identification of such sequences in a scaffold enables us to assign it to a subgenomic lineage. First, k-mer spectra for each scaffold was generated using *jellyfish*. After importing these k-mer spectra into R, tables are created for each homoeologous pair, with each column containing the counts for each given k-mer observed in the pair. These counts are then filtered for abundance; k-mers must appear in a given scaffold at least

75 times, and pairs must share more than 75 orthologs. A second filter is applied to keep only k-mers that are doubly distributed in one counterpart of the pair over the other, to remove k-mers that are not ancestral. To control for varying scaffold lengths, and therefore total k-mer counts, all k-mer counts passing the previous two filters were converted to binary ratios. Scaffolds were then hierarchically clustered according to presence or absence of k-mers and organised into a cladogram, containing two clusters representing scaffolds assigned to subgenome A or subgenome B. The cladogram was plotted using R's base plotting library. Scaffolds assigned to pairs but not phased by the k-mer method were assigned to a subgenome based on the k-mer based assignment of its counterpart.

Sequence divergence

Within subgenomes

Allelic divergence within subgenomes was estimated in the following way. For each subgenome, a subset of reads exclusive to a given subgenome was extracted from the total mapped HiFi reads. A k-mer distribution was then generated for these reads, and a histogram file generated. These histograms were interpreted with *Genomescope* [38], which predicted the amount of allelic divergence between each subgenomes' alleles.

Between subgenomes

Sequence divergence between subgenomes was calculated for both whole sequence and CDS regions. For whole sequence similarity, subgenome A and subgenome B assigned scaffolds were aligned with *minimap2* [97] using the *–ax asm10* preset, and divergence calculated from the number of aligned bases divided by the number of

concordant bases. For CDS alignment, the same method was applied using paired CDS extracted during synteny analysis.

Synteny analysis

We used *MCScan (Python)* [90, 91] to detect synteny between phased subgenomes. Subgenome specific CDS annotations generated from Iso-Seq transcript data and gene predictions identified by *MAKER3* were extracted and transformed into bed format. *LAST* aligner was then used to detect orthologs of each feature on the opposing subgenome. Identified syntenic pairs were grouped into blocks based on proximity, producing an anchor file. We then converted this anchor file into a pseudo-bed format containing coordinates of start and stop positions of each syntenic block. These coordinates were used to plot the synteny using R. Scripts are available in our Zenodo repository (doi: 10.5281/zenodo.7858245) and also upon request.

92. Altschul SF, Gish W, Miller W, Myers EW, Lipman DJ. Basic local alignment search tool. J Mol Biol. 1990;215: 403–410. doi:10.1016/S0022-2836(05)80360-2

93. Bernt M, Donath A, Jühling F, Externbrink F, Florentz C, Fritzsch G, et al. MITOS: improved de novo metazoan mitochondrial genome annotation. Mol Phylogenet Evol. 2013;69: 313–319. doi:10.1016/j.ympev.2012.08.023

94. Li H, Handsaker B, Wysoker A, Fennell T, Ruan J, Homer N, et al. The Sequence Alignment/Map format and SAMtools. Bioinformatics. 2009;25: 2078–2079. doi:10.1093/bioinformatics/btp352

95. Gurevich A, Saveliev V, Vyahhi N, Tesler G. QUAST: quality assessment tool for genome assemblies. Bioinformatics. 2013;29: 1072–1075. doi:10.1093/bioinformatics/btt086

96. Marçais G, Delcher AL, Phillippy AM, Coston R, Salzberg SL, Zimin A. MUMmer4: A fast and versatile genome alignment system. PLoS Comput Biol. 2018;14: e1005944. doi:10.1371/journal.pcbi.1005944

97. Li H. Minimap2: pairwise alignment for nucleotide sequences. Bioinformatics. 2018;34: 3094–3100. doi:10.1093/bioinformatics/bty191
