# Supplementary material for: Phased chromosome-scale genome assembly of an asexual, allopolyploid root-knot nematode reveals complex subgenomic structure
Source: PLoS One. 2024 Jun 6;19(6):e0302506. doi: 10.1371/journal.pone.0302506 (PMC11156385; doi:10.1371/journal.pone.0302506)
Supplement: S2 File — (PDF) [file pone.0302506.s002.pdf]

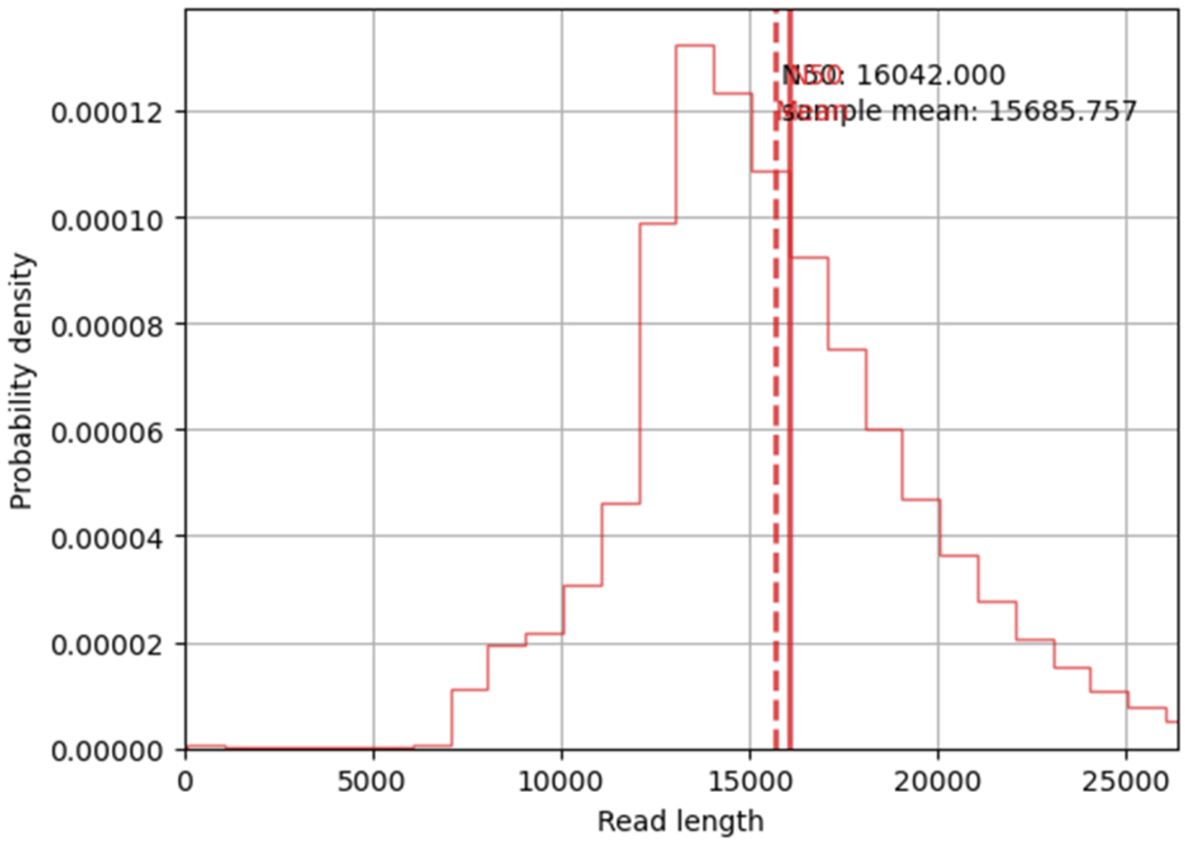

Supplementary Figure 1: Length and quality statistics of concatenated and quality controlled PacBio HiFi libraries. X axis is read length. Y axis is probability density.

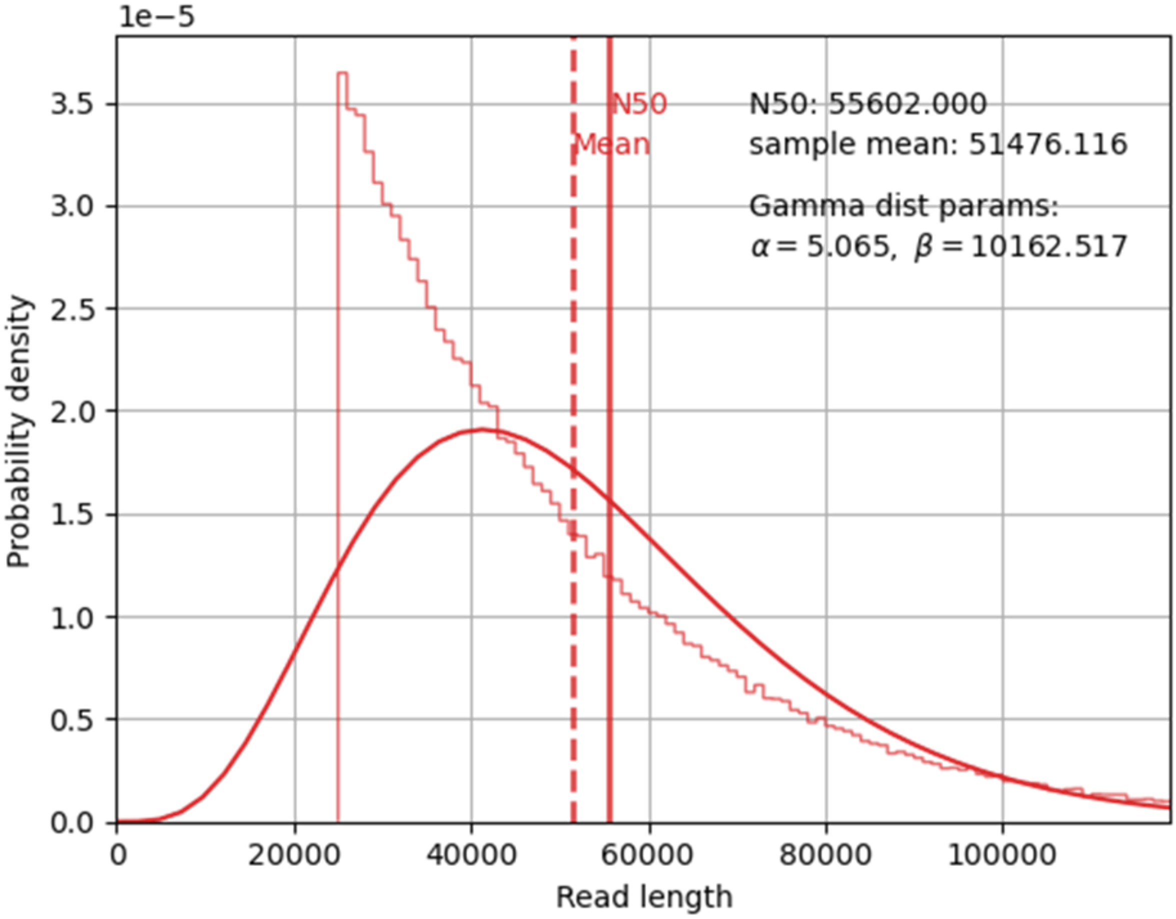

Supplementary Figure 2: Length and quality statistics of concatenated and quality controlled Oxford Nanopore libraries. X axis is read length. Y axis is probability density.

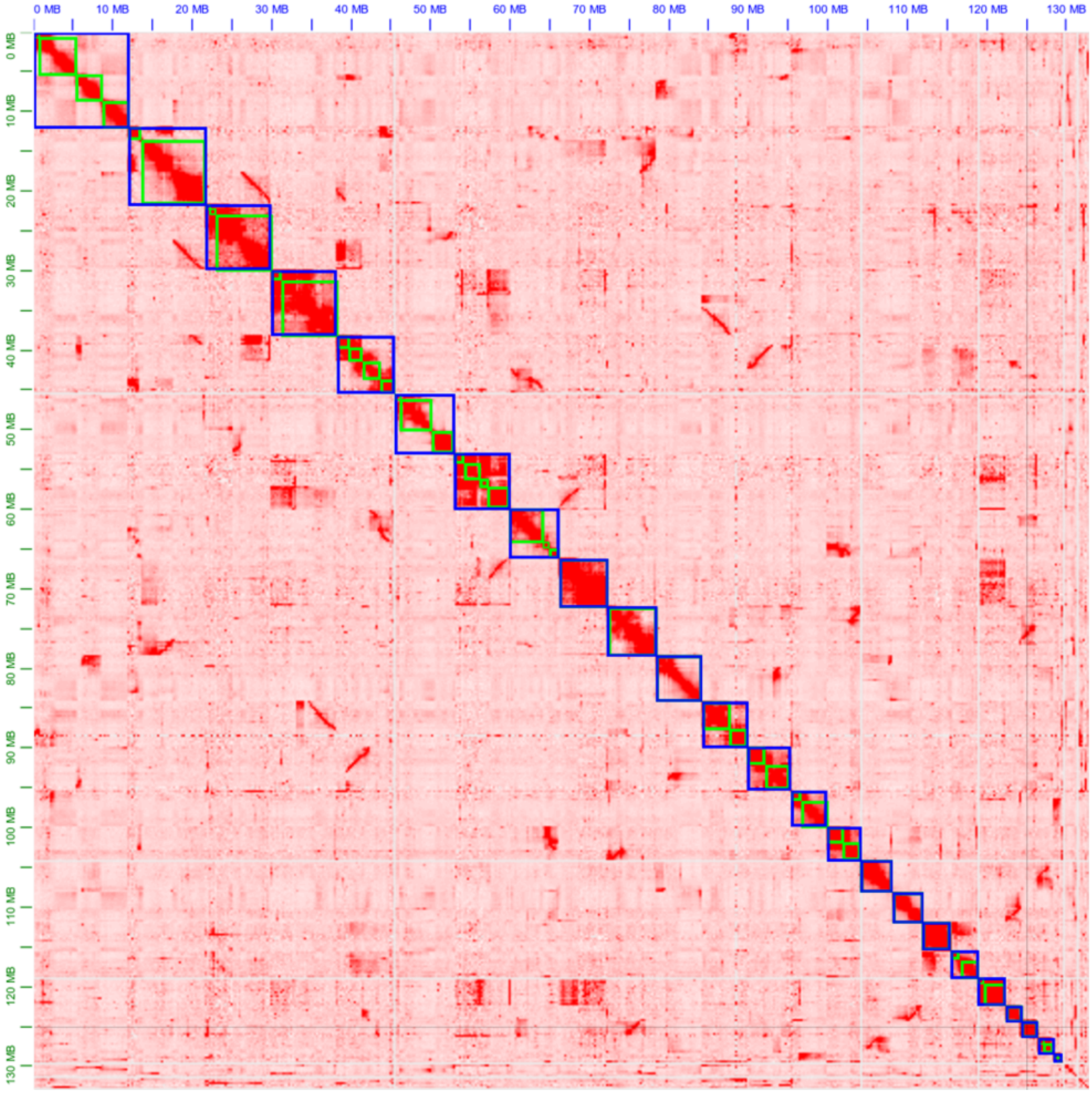

Supplementary Figure 3: Chromatin contact map following Hi-C scaffolding and manual curation. Ordered according to length. Generated by *Juicebox.*

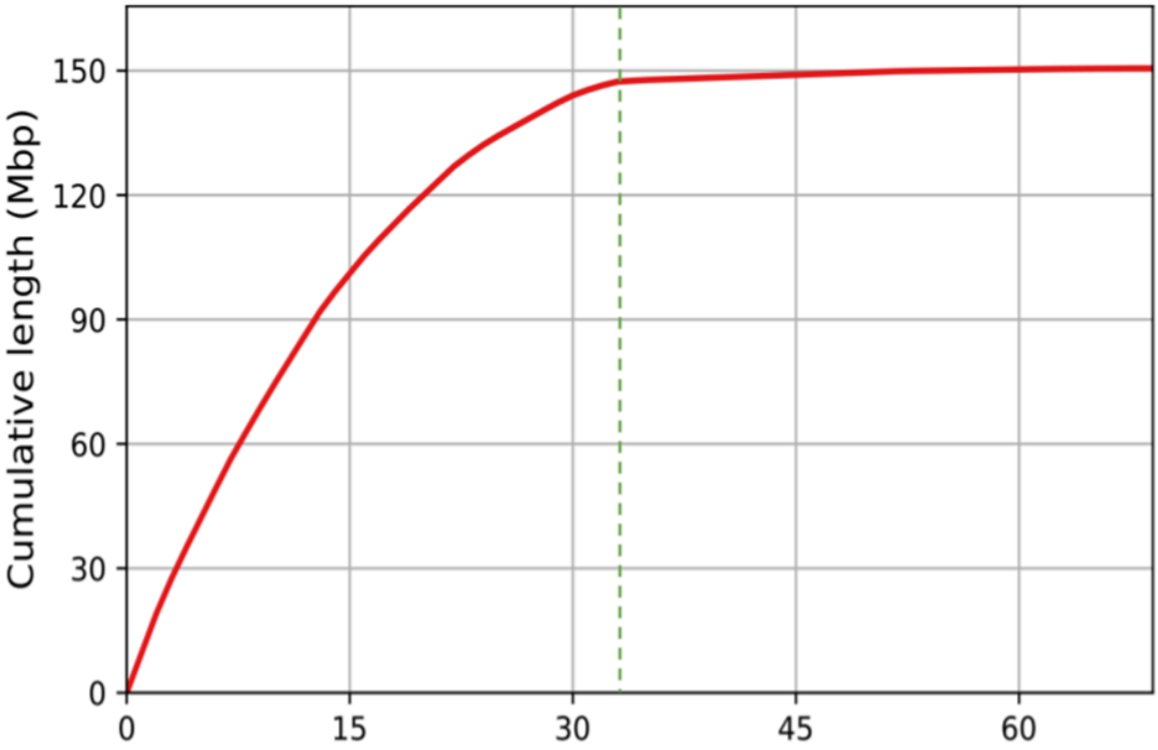

Supplementary Figure 4: Total cumulative length over number of scaffolds. Generated using *QUAST*. X axis is number of scaffolds. Y axis is cumulative length in megabases. Green dashed line indicates the end of scaffold 33.

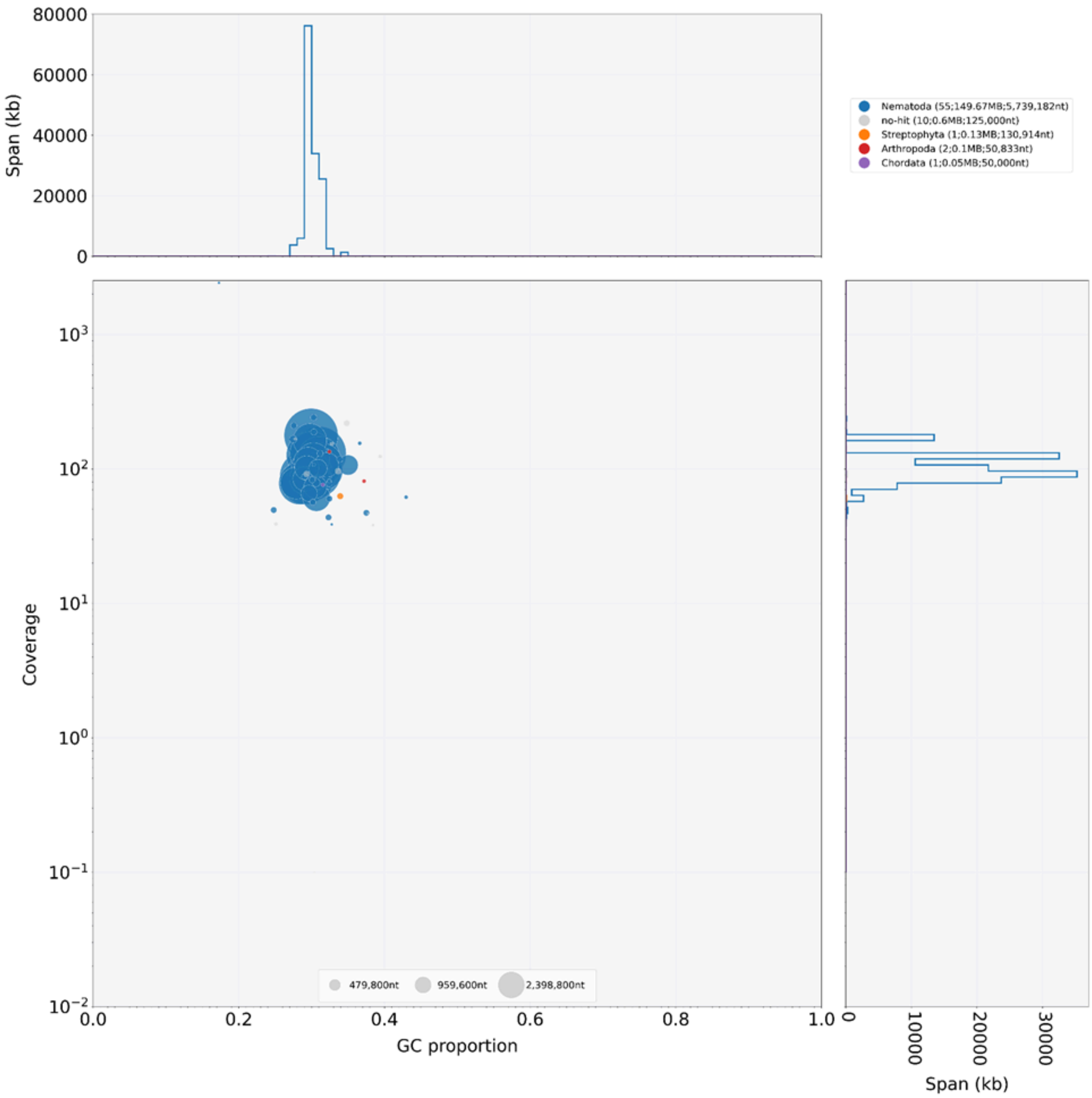

Supplementary Figure 5: Blobplot of contamination in our *Meloidogyne javanica* assembly. The x-axis represents the GC proportion, and the y-axis the coverage depth of sequences in the assembly. The size and colour of each blob indicate the relative abundance and taxonomic identity of the contaminating organisms according to the key.

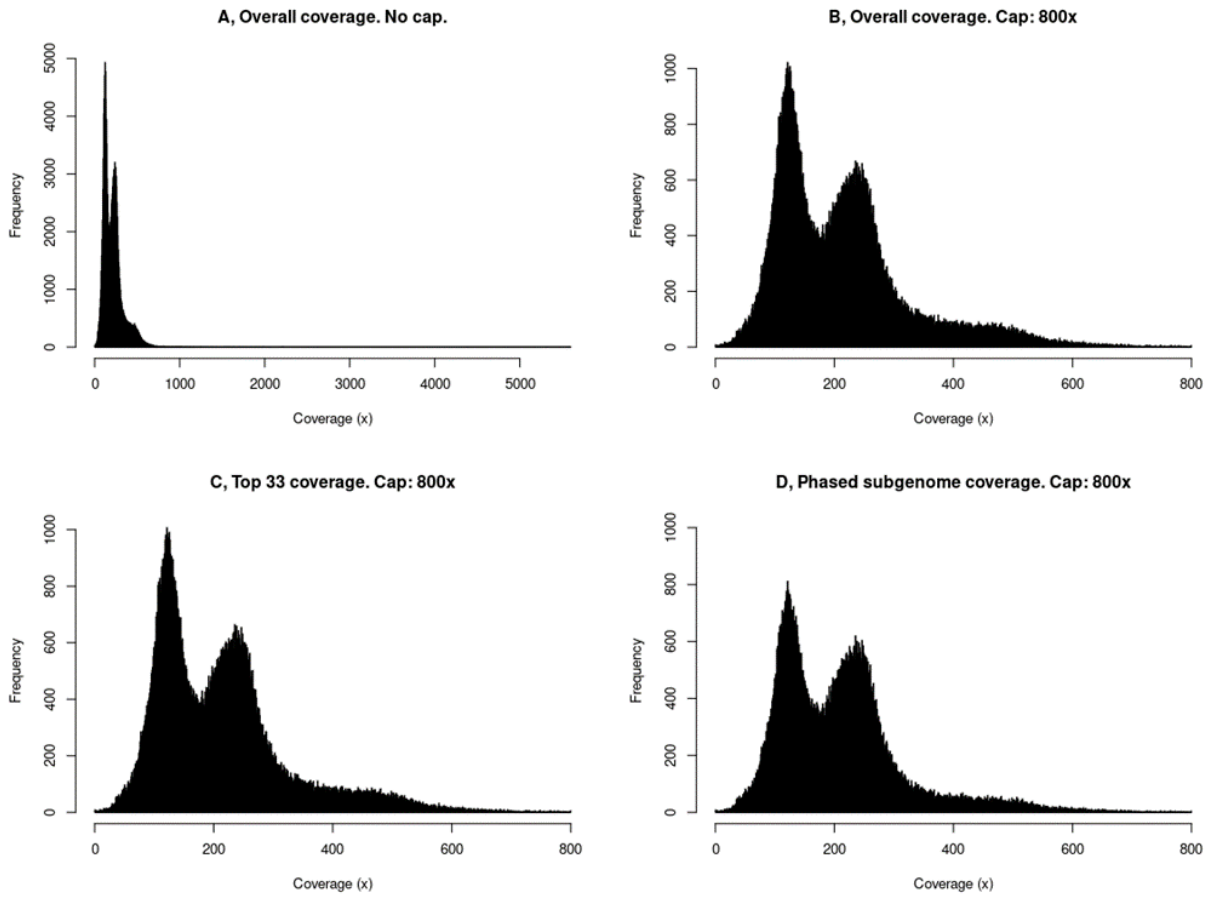

Supplementary Figure 6: Assembly-wide distributions of coverage depth. A, Distribution of coverage depth at all bases in the assembly. **B**, Distribution of coverage depth of all bases in the assembly, capped at 800x. **C**, Distribution of coverage of all bases in the longest 33 scaffolds, capped at 800x. **D**, Distribution of coverage of all bases in scaffolds phased to a subgenome, capped at 800x. Breaks = 1000. Bin size = 0.8.

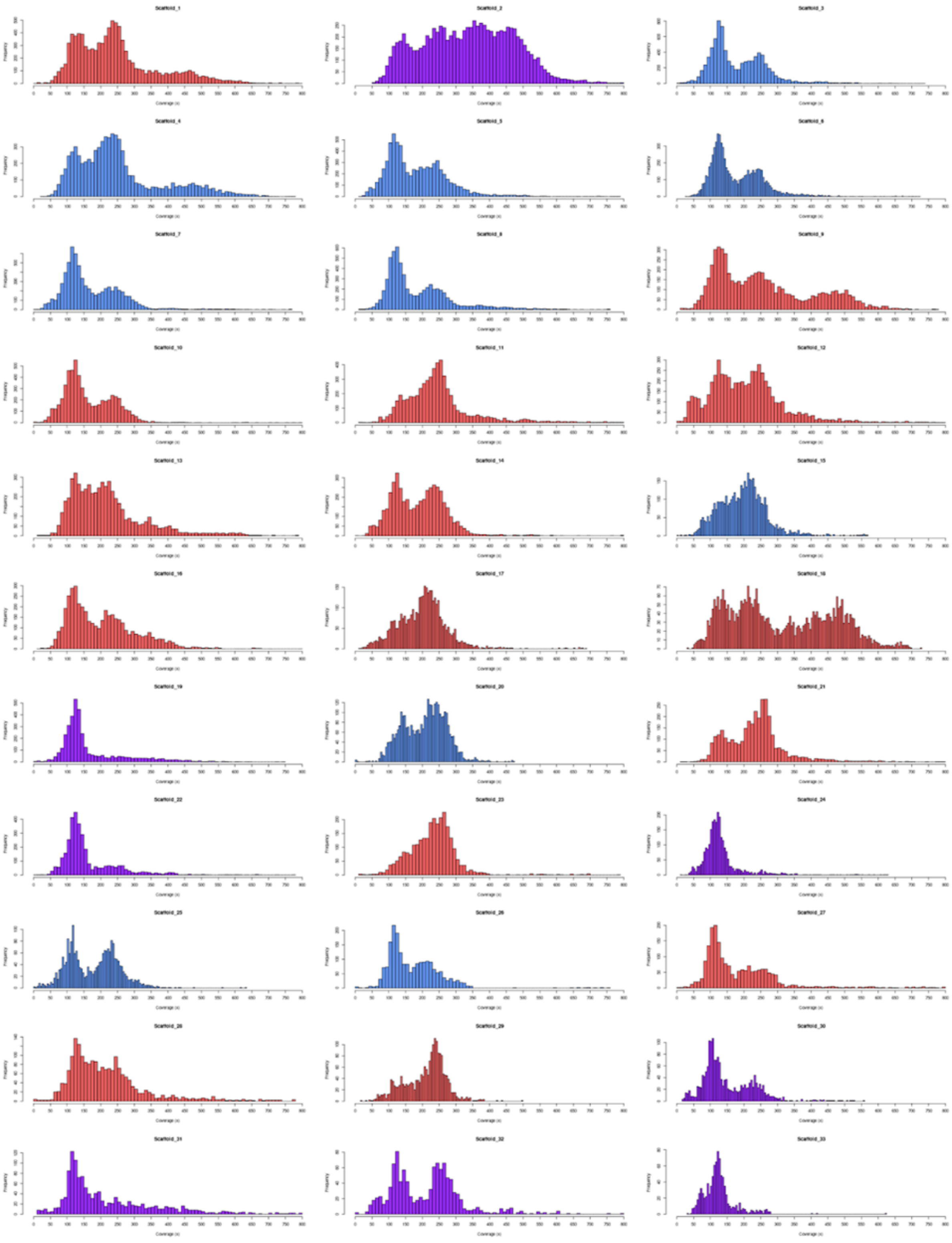

Supplementary Figure 7: Scaffold-level distributions of coverage depth frequencies. Subgenome A in blue, subgenome B in red, and unphased scaffolds in purple. X-axis shows coverage depth, y-axis shows frequency. Breaks = 80. Bin size = 10.

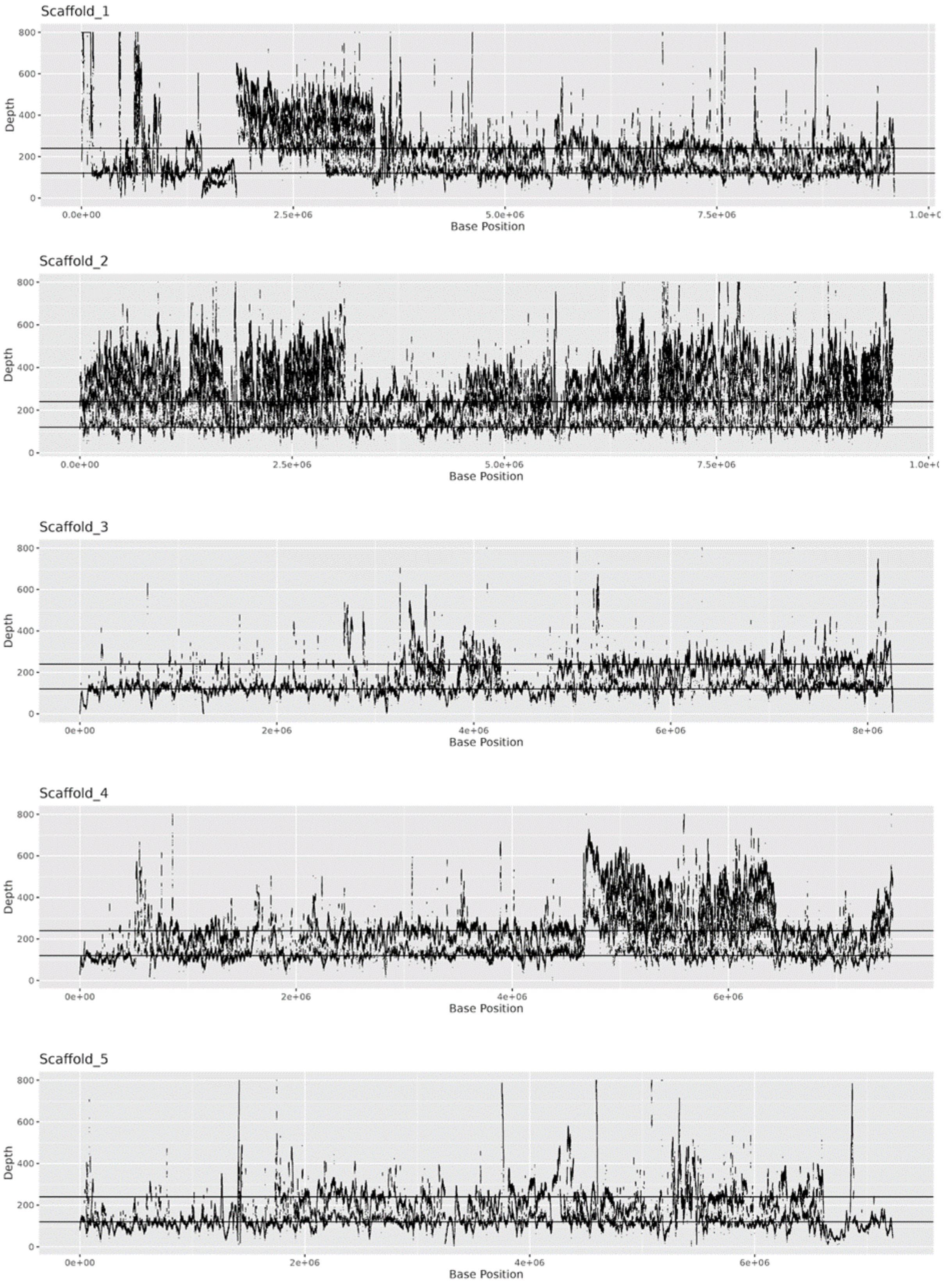

Supplementary Figure 8a: Coverage depth of individual bases across scaffolds 1-5. Horizontal lines mark 120x and 240x coverage depth, corresponding to peaks seen in Supplementary Figure 7.

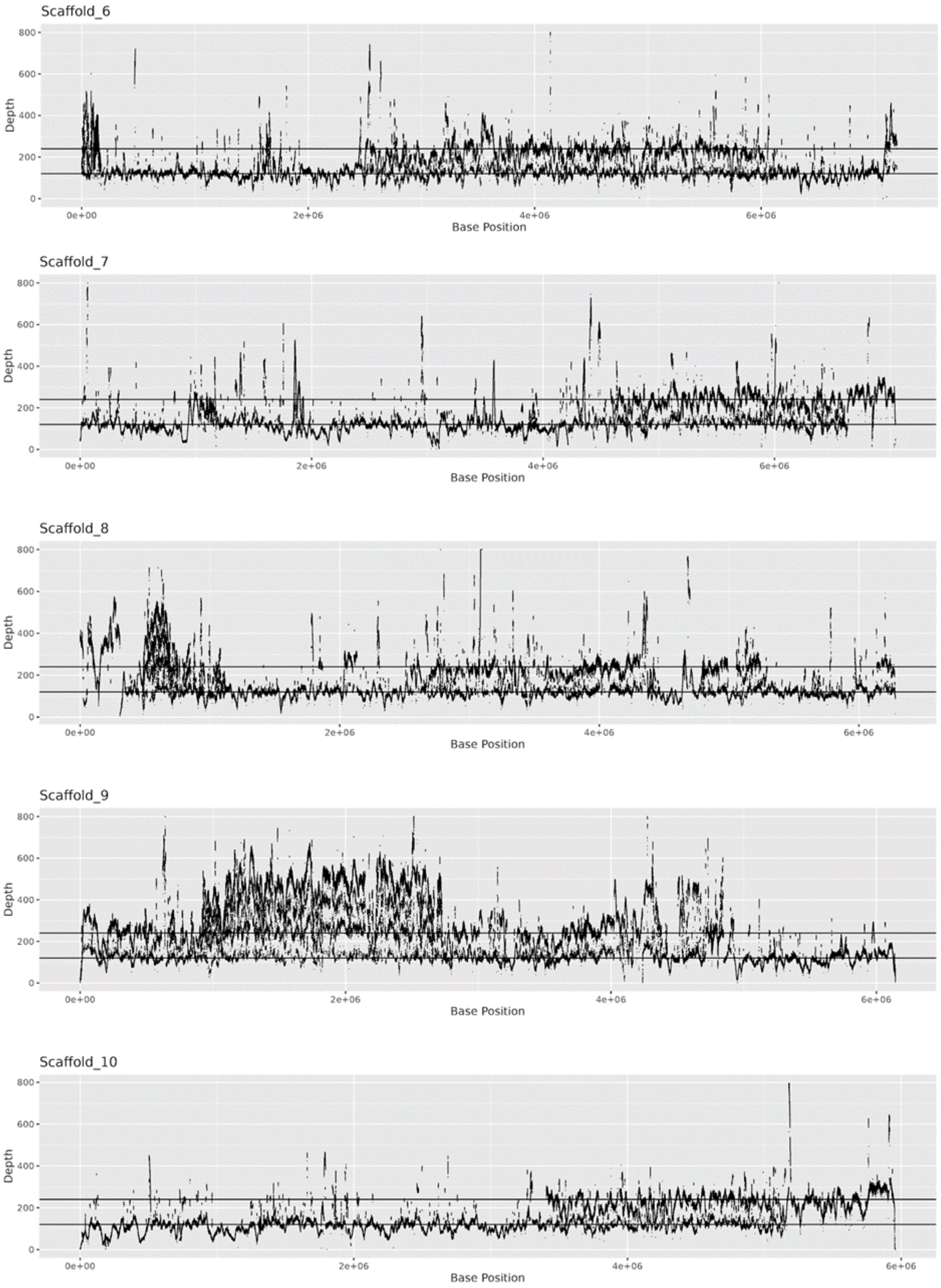

Supplementary Figure 8b: Coverage depth of individual bases across scaffolds 6-10. Horizontal lines mark 120x and 240x coverage depth, corresponding to peaks seen in Supplementary Figure 7.

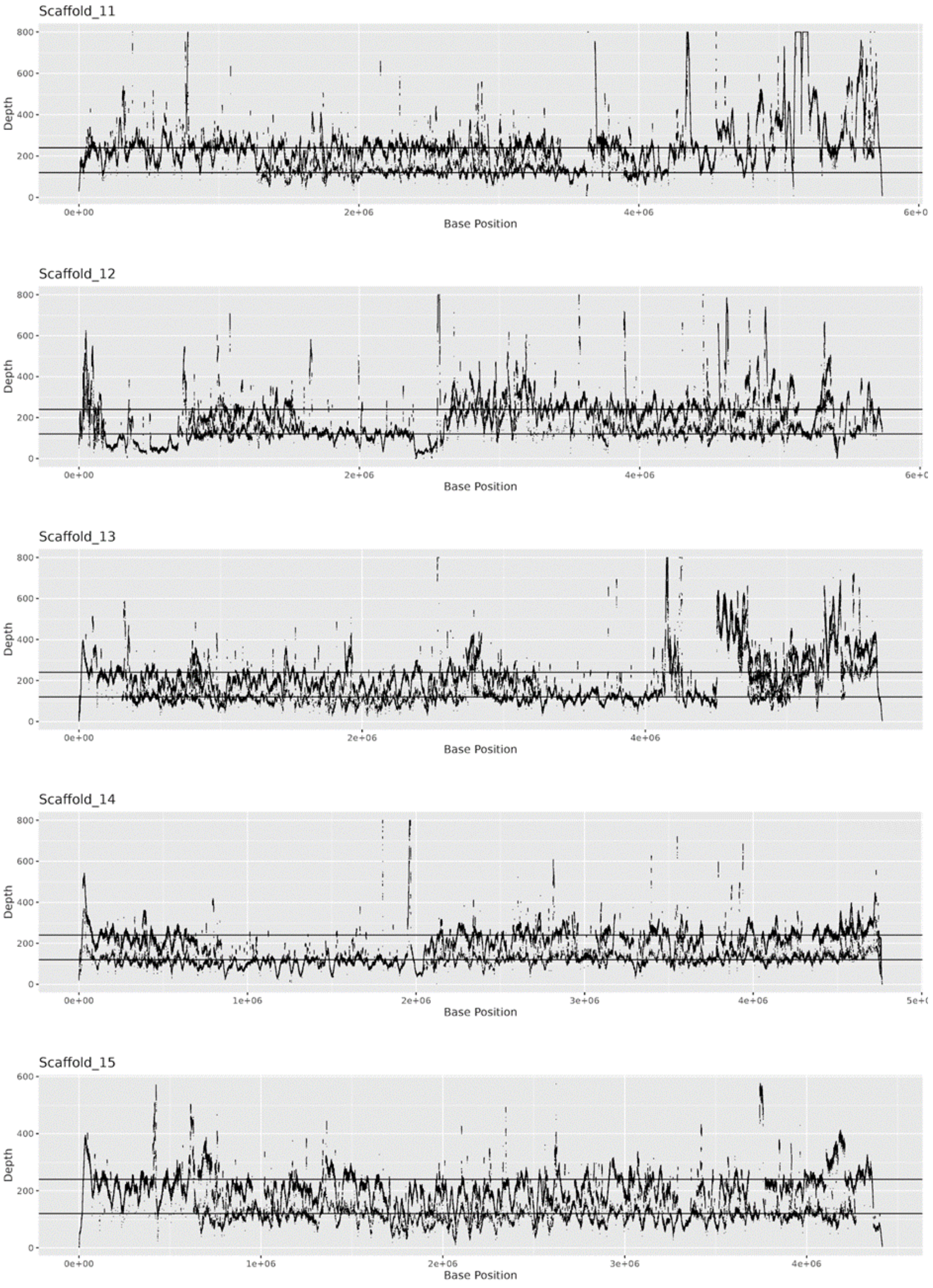

Supplementary Figure 8c: Coverage depth of individual bases across scaffolds 11-15. Horizontal lines mark 120x and 240x coverage depth, corresponding to peaks seen in Supplementary Figure 7.

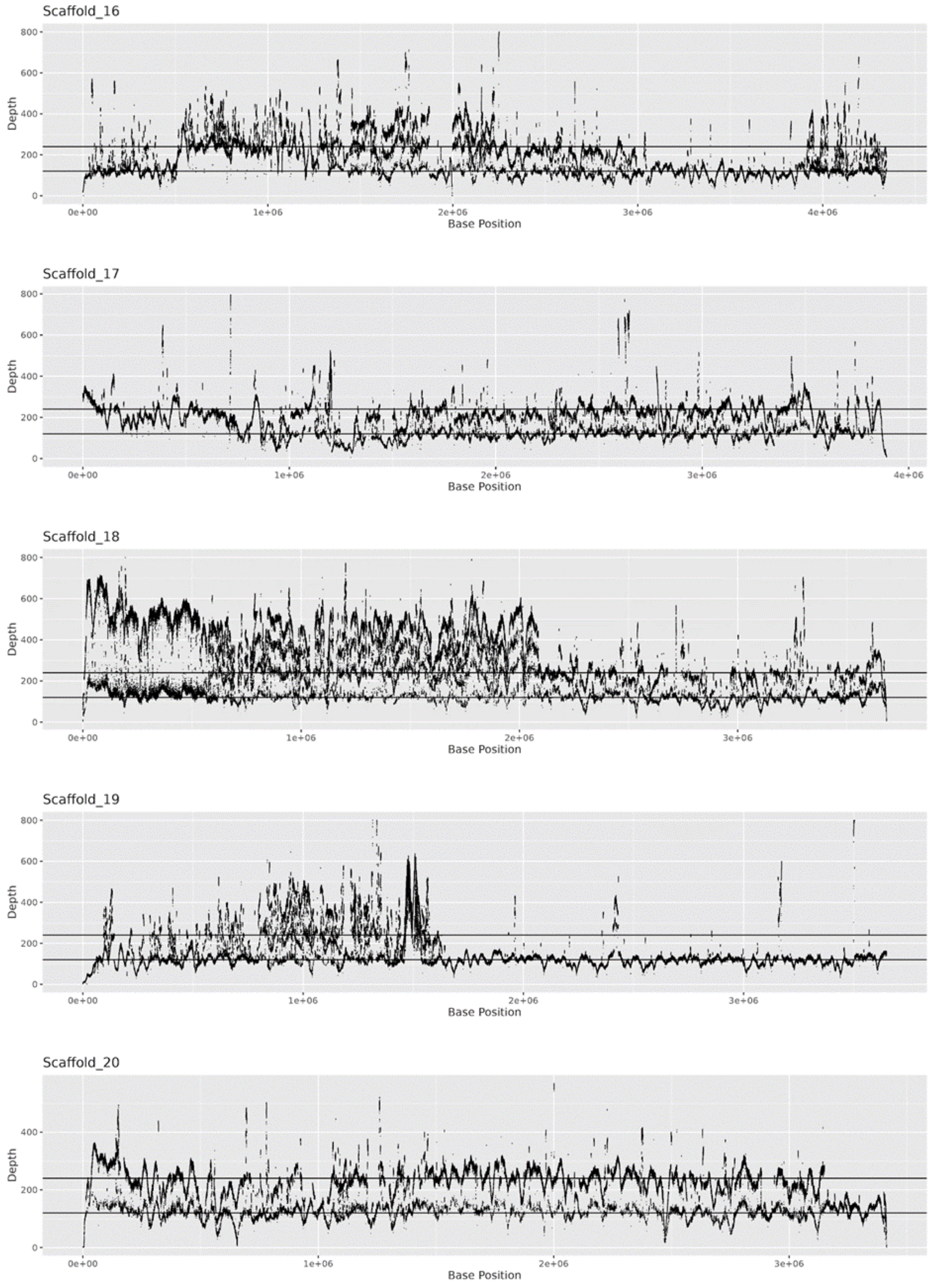

Supplementary Figure 8d: Coverage depth of individual bases across scaffolds 16-20. Horizontal lines mark 120x and 240x coverage depth, corresponding to peaks seen in Supplementary Figure 7.

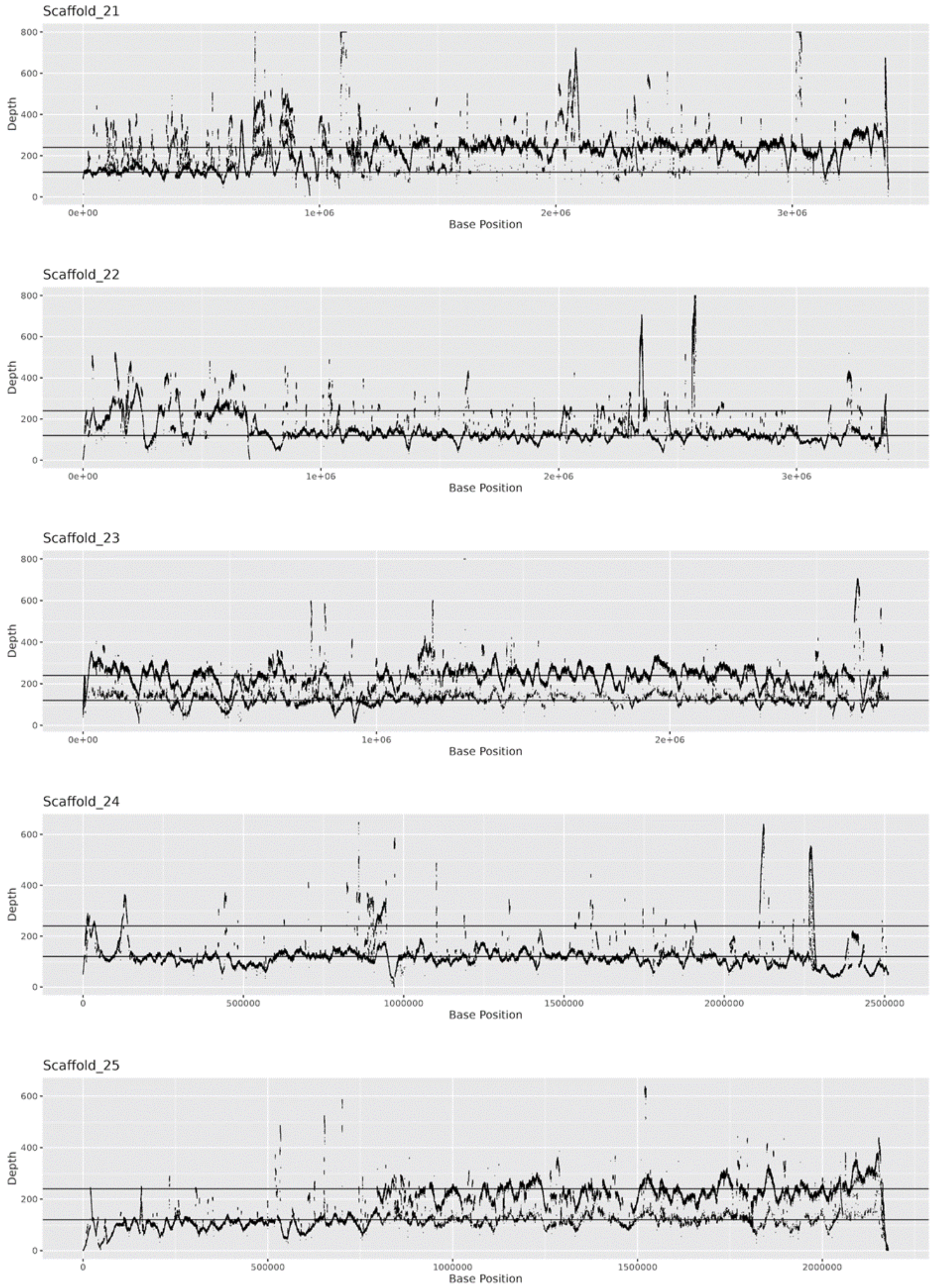

Supplementary Figure 8e: Coverage depth of individual bases across scaffolds 21-25. Horizontal lines mark 120x and 240x coverage depth, corresponding to peaks seen in Supplementary Figure 7.

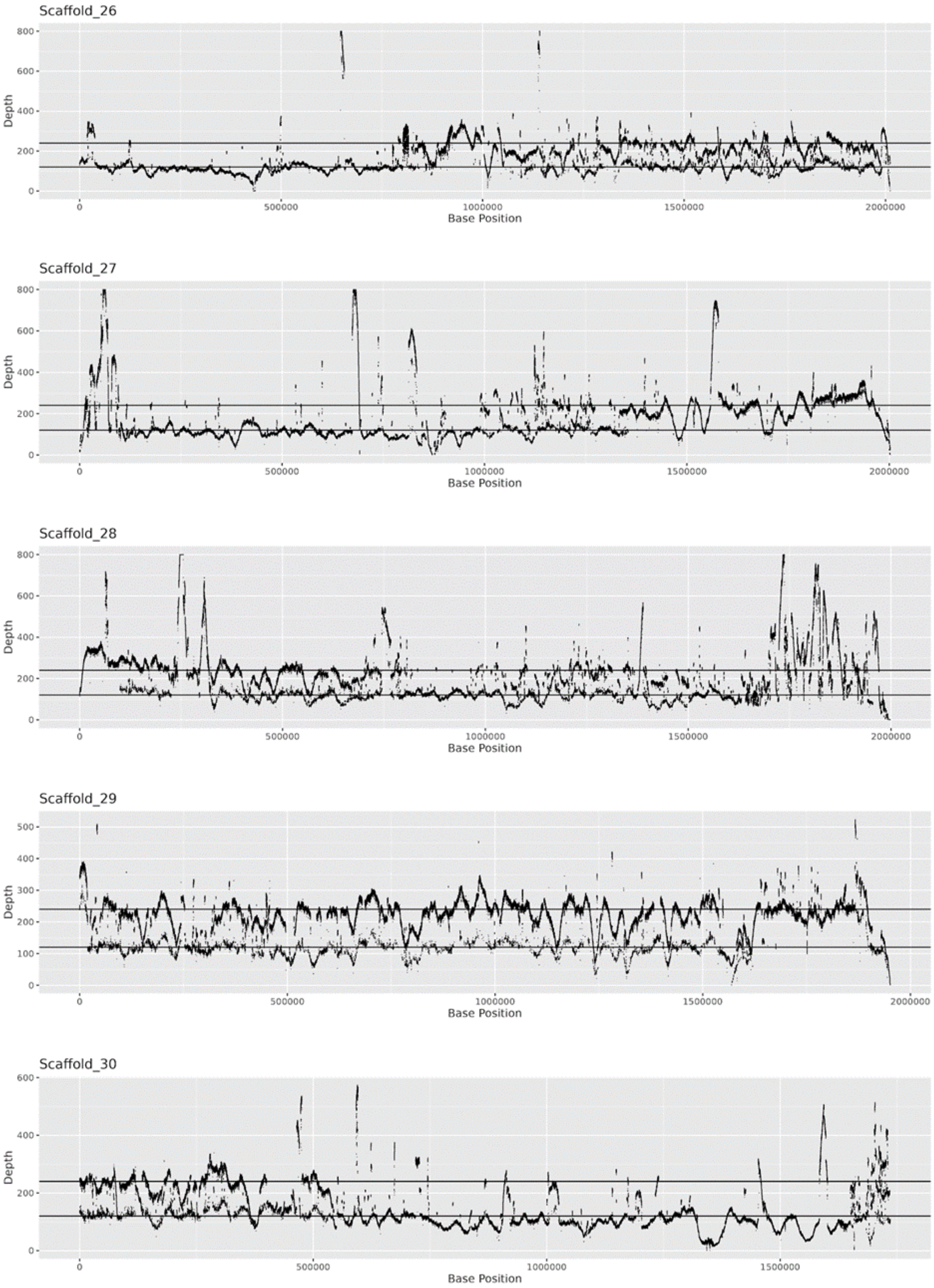

Supplementary Figure 8f: Coverage depth of individual bases across scaffolds 26-30. Horizontal lines mark 120x and 240x coverage depth, corresponding to peaks seen in Supplementary Figure 7.

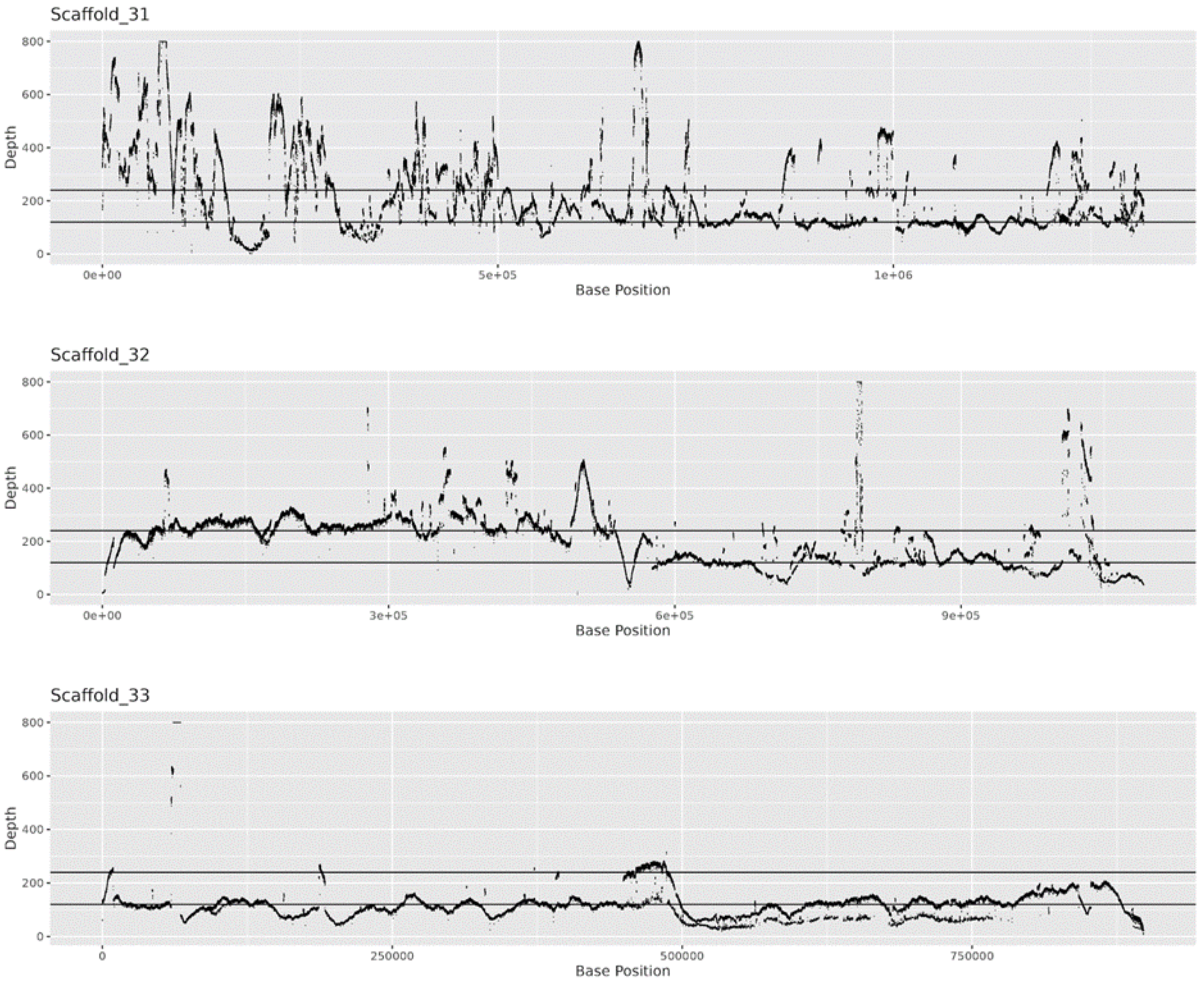

Supplementary Figure 8g: Coverage depth of individual bases across scaffolds 31-33. Horizontal lines mark 120x and 240x coverage depth, corresponding to peaks seen in Supplementary Figure 7.

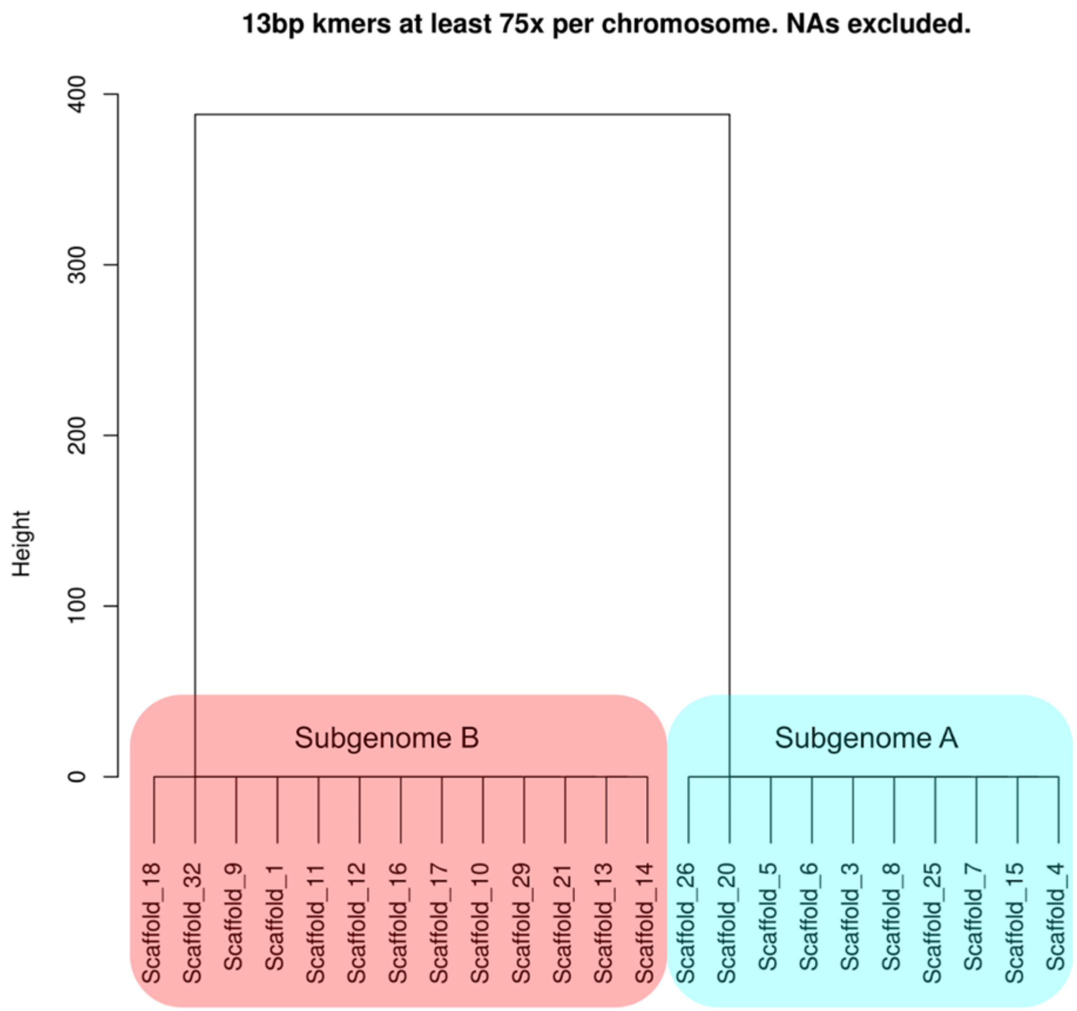

Supplementary Figure 9: Subgenome-phased dendrogram of scaffolds. Generated in *R* using hierarchical clustering based on presence of ancestral k-mers. Scaffolds in blue are assigned to subgenome A. Scaffolds in red are assigned to subgenome B.
