# Supplementary material for: Phased chromosome-scale genome assembly of an asexual, allopolyploid root-knot nematode reveals complex subgenomic structure
Source: PLoS One. 2024 Jun 6;19(6):e0302506. doi: 10.1371/journal.pone.0302506 (PMC11156385; doi:10.1371/journal.pone.0302506)
Supplement: S3 File — (PDF) [file pone.0302506.s003.pdf]

Supplementary Table 1: Descriptive statistics of assembly and its contemporaries.

Accession	Species	Year	Scaffolds	Assembly size (mbp)	Nuclear DNA Content (mbp)	N50 (kbp)	CEGMA % (complete: C, partial: P)	BUSCO v5 % (complete: C [single: S, duplicated: D], fragmented: F, missing: M)	GC %
GCA_034785575.1	*M. javanica** (Hull)	2023	69	150.5	NA	5,793	C :93.95 (1.88) P: 95.56	C:69.5 [S:37.3%, D:32.2%], F:13.7%, M:16.8%	30.1
GCA_003693625.1	*M. javanica* (Hull)	2017	34,394	142.6	NA	14.1	C :89.52 (2.71) P: 95.16	C:70.2% [S:30.2, D:40.0%], F:13.3%, M:16.5%	30.2
GCA_900003945.1	*M. javanica* (Avignon)	2017	31,341	235.8	297+- 27	10.4	C: 92.74 (3.68) P :95.56	C:70.2% [S:14.9%, D:55.3%], F:14.1%, M:15.7%	30
GCA_014132215.1	*M. incognita* (Morelos)	2017	12,091	183.5	189 +- 15	38.6	C: 94.76 (2.93) P :96.77	C:71.7% [S:18.8%, D:52.9%], F:11.4%, M:16.9%	29.8
GCA_003693645.1	*M. incognita* (Hull)	2017	33,735	122	NA	16.5	C: 82.66 (2.34) P :89.52	C:61.6% [S:29.4%, D:32.2%], F:16.9%, M:21.5%	30.6
GCA_000172435.1	*M. hapla* (VW9)	2008	1,523	53.6	121 +- 3	83.6	C :93.55 (1.19) P: 95.56	C:66.7[S:65.9%, D:0.8%], F:16.1%, M:17.2%	27.4
GCA_003693605.1	*M. floridensis (SJF1)*	2018	9,134	74.9	NA	13.3	C: 77.42 (1.71) P: 83.87	C:58.4% [S:53.3%, D:5.1%], F:17.3%, M:24.3%	30.2
GCA_902706615.1	*M. luci* (SI-Smartno)	2020	327	209.2	NA	1,712	C: 95.56 (2.92) P :96.77	C:73.7% [S:14.9%, D:58.8%], F:11.0%, M:15.3%	30.2
GCA_903994135.1	*M. enterolobii* (Swiss)	2021	4,437	240	275 +- 19	143	C: 94.76 (3.30) P: 96.77	C:73.7% [S:13.3%, D:60.4%], F:10.6%, M:15.7%	30
GCA_900003985.1	*M. arenaria (Guadeloupe)*	2017	26,196	258.1	304 +- 9	16.5	C :94.76 (3.66) P :95.56	C:70.5% [S:12.9%, D:57.6%], F:13.3%, M:16.2%	30
GCA_003133805.1	*M. arenaria (A2-0)*	2019	2,224	284.05	NA	204.6	C: 94.76 (3.57) P :96.77	C:72.6% [S:17.3%, D:55.3%], F:12.2%, M:15.2%	30
GCA_002778205.2	*M. graminicola (IARI)*	2022	4,304	38.18	NA	20.4	C: 84.27 (1.34) P: 90.73	C:65.1% [S:56.1%, D:9.0%], F:13.3%, M:21.6%	23.05

Supplementary Table 2: Summary of longest 33 scaffolds of diploid assembly of *M. javanica*.

Scaffold Number	Length (bp)	Mean coverage (x)	Phase assignment (A, B, or unphased)	Comments
1	9595054	245	B	Some collapse.
2	9577269	337	U	Extensive collapse.
3	8254935	181	A	
4	7520906	251	A	Some collapse.
5	7228418	184	A	
6	7199321	177	A	
7	7044505	169	A	
8	6281301	186	A	
9	6143287	260	B	Some collapse.
10	5957753	164	B	
11	5739182	247	B	
12	5730042	205	B	
13	5673700	219	B	
14	4766193	189	B	
15	4415488	196	A	
16	4345108	204	B	Some collapse.
17	3893252	201	B	
18	3682237	314	B	Some collapse.
19	3649086	168	U	Some collapse.
20	3413746	207	A	
21	3407168	264	B	
22	3386470	162	A	Low coverage, possibly single copy. Phased manually.
23	2744999	231	B	Phased manually.
24	2512086	131	U	Low coverage, possibly single copy.
25	2178917	178	A	
26	2011659	171	A	
27	2002407	183	B	Phased manually.
28	1997981	214	B	Phased manually.
29	1951323	210	B	
30	1735511	151	U	
31	1316575	219	U	

32	1091457	211	B	
33	898067	125	B	Low coverage, possibly single copy. Phased manually.

[1] Here collapse refers to regions of a scaffold with more than 2 copies predicted based on read depth.

Supplementary Table 3: Tabular breakdown of repeat annotation from *RepeatMasker*.

	Number of elements	Length occupies (bp)	Percentage of sequence (%)
**Retroelements**	11491	7443793	4.94
SINEs	0	0	0.00
Penelope	0	0	0.00
LINEs	663	657807	0.44
CRE/SLACS	0	0	0.00
L2/CR1/Rex	588	633246	0.42
R1/LOA/Jockey	0	0	0.00
R2/R4/NeSL	0	0	0.00
RTE/bOV-b	0	0	0.00
L1/CIN4	34	3868	0.00
LTR Elements	10828	6785986	4.51
BEL/Pao	2888	3074846	2.04
Ty1/Copia	0	0	0.00
Gypsy/DIRS1	3129	2254025	1.50
**DNA Transposons**	7992	5674442	3.77
Hobo-Activator	1547	261731	0.17
Tc1-IS630-Pogo	1847	601027	0.40
En-Spm	0	0	0.00
MuDR-IS905	0	0	0.00
PiggyBac	0	0	0.00
Tourist/Harbinger	0	0	0.00
Other (Mirage, P-element, Transib)	0	0	0.00
**Rolling-circles**	4285	1796873	1.19
**Unclassified**	129631	25417075	16.88
**Total interspersed repeats**	-	38535310	25.60
**Small RNA**	1092	1514780	1.01
**Satellites**	526	59215	0.04
**Simple repeats**	55163	2683708	1.78
**Low complexity**	24100	1270259	0.84

Supplementary Table 4: Full table of results from structural annotation with MAKER3.

Metric	Count
Number of genes	22433
Number of mrnas	22433
Number of mrnas with utr both sides	2811
Number of mrnas with at least one utr	12486
Number of cds	22433
Number of exons	227617
Number of five_prime_utrs	10044
Number of three_prime_utrs	5253
Number of exon in cds	224453
Number of exon in five_prime_utr	12537
Number of exon in three_prime_utr	5783
Number of intron in cds	202020
Number of intron in exon	205184
Number of intron in five_prime_utr	2493
Number of intron in three_prime_utr	530
Number of single exon gene	91
Number of single exon mrna	91
mean mrnas per gene	1
mean cds per mrna	1
mean exons per mrna	10.1
mean five_prime_utrs per mrna	0.4
mean three_prime_utrs per mrna	0.2
mean exons per cds	10
mean exons per five_prime_utr	1.2
mean exons per three_prime_utr	1.1
mean introns in cds per mrna	9
mean introns in exons per mrna	9.1
mean introns in five_prime_utrs per mrna	0.1
mean introns in three_prime_utrs per mrna	0
Total gene length	70550747
Total mrna length	70550747
Total cds length	29119128
Total exon length	30170066
Total five_prime_utr length	525976
Total three_prime_utr length	524962

Total intron length per cds	40117619
Total intron length per exon	40585865
Total intron length per five_prime_utr	365056
Total intron length per three_prime_utr	85815
mean gene length	3144
mean mrna length	3144
mean cds length	1298
mean exon length	132
mean five_prime_utr length	52
mean three_prime_utr length	99
mean cds piece length	129
mean five_prime_utr piece length	41
mean three_prime_utr piece length	90
mean intron in cds length	198
mean intron in exon length	197
mean intron in five_prime_utr length	146
mean intron in three_prime_utr length	161
Longest genes	85925
Longest mrnas	85925
Longest cds	58605
Longest exons	36616
Longest five_prime_utrs	1124
Longest three_prime_utrs	2255
Longest cds piece	36616
Longest five_prime_utr piece	871
Longest three_prime_utr piece	2230
Longest intron into cds part	46014
Longest intron into exon part	46014
Longest intron into five_prime_utr part	6288
Longest intron into three_prime_utr part	5958
Shortest genes	24
Shortest mrnas	24
Shortest cds	6
Shortest exons	2
Shortest five_prime_utrs	1
Shortest three_prime_utrs	1
Shortest cds piece	1
Shortest five_prime_utr piece	1

Shortest three_prime_utr piece	1
Shortest intron into cds part	5
Shortest intron into exon part	5
Shortest intron into five_prime_utr part	5
Shortest intron into three_prime_utr part	5

Supplementary Table 5: List of pairs that share CDS and amount of shared links.

Reference Scaffold	Target Scaffold	Shared CDS
2	13	46
3	11	284
4	14	307
5	12	182
6	19	216
6	1	206
7	10	206
8	16	273
8	33	162
8	29	58
8	28	49
9	5	102
13	15	351
17	25	112
18	26	63
20	23	214
20	32	120
24	30	20
25	27	64

Supplementary Table 6: Validation of homoeologous pairings.

Scaffold number	Orthology	MASH	BUSCO	Consensus homoeologous counterpart
1	6	6	6	6
2	13	-	52	13
3	11	11	7	11
4	14	14	6	14
5	12	12	9	12
6	21	21	-	21
7	10	10	-	10
8	16	16	-	16
9	5	-	7	5
10	7	7	3	7
11	3	3	5	3
12	5	5	15	5
13	15	15	45	15
14	4	4	-	4
15	13	13	8	13
16	8	8	25	8
17	25	25	26	25
18	26	-	21	26
19	6	6	30	6
20	23	23	6	23
21	6	6	-	6
22	10	10	-	10
23	20	20	-	20
24	30	-	-	30
25	17	27	-	17
26	18	-	49	18
27	25	-	-	25
28	8	-	8	8
29	8	-	-	8
30	24	-	-	24

31	6	-	-	6
32	20	-	-	20
33	8	-	-	8

Supplementary Table 7: Descriptive statistics of each phased subgenome

	Subgenome A	Subgenome B
**Number of sequences**	11	17
**Length**	58,935,666	69,619,210
**N50**	7,199,321	5,673,700
**GC%**	29.8	30.27
**Largest contig**	8,254,935	9,595,054
